# Preconception indicators and associations with health outcomes reported in UK routine primary care data: a systematic review

**DOI:** 10.3399/BJGP.2024.0082

**Published:** 2025-01-14

**Authors:** Danielle Schoenaker, Elizabeth M Lovegrove, Emma H Cassinelli, Jennifer Hall, Majel McGranahan, Laura McGowan, Helen Carr, Nisreen A Alwan, Judith Stephenson, Keith M Godfrey

**Affiliations:** School of Human Development and Health, MRC Lifecourse Epidemiology Centre, University of Southampton; National Institute for Health and Care Research (NIHR) Southampton Biomedical Research Centre, University of Southampton; University Hospital Southampton NHS Foundation Trust, Southampton.; Primary Care Research Centre, University of Southampton, Southampton.; Centre for Public Health, Queen’s University Belfast, Belfast.; University College London Elizabeth Garrett Anderson Institute for Women’s Health, University College London, London.; Warwick Medical School, University of Warwick, Coventry.; Centre for Public Health, Queen’s University Belfast, Belfast.; NHS Surrey Heartlands Integrated Care Partnership, Guildford, Surrey.; School of Primary Care, Population Sciences and Medical Education, University of Southampton; NIHR Southampton Biomedical Research Centre, University of Southampton; University Hospital Southampton NHS Foundation Trust; NIHR Applied Research Collaboration Wessex, Southampton.; University College London Elizabeth Garrett Anderson Institute for Women’s Health, University College London, London.; School of Human Development and Health, MRC Lifecourse Epidemiology Centre, University of Southampton; National Institute for Health and Care Research (NIHR) Southampton Biomedical Research Centre, University of Southampton; University Hospital Southampton NHS Foundation Trust, Southampton.

**Keywords:** general practice, preconception care, pregnancy, pregnancy outcomes, pre-pregnancy care, primary care

## Abstract

**Background:**

Routine primary care data may be a valuable resource for preconception health research and to inform the provision of preconception care.

**Aim:**

To review how primary care data could provide information on the prevalence of preconception indicators and examine associations with maternal and offspring health outcomes.

**Design and setting:**

Systematic review of observational studies using UK routine primary care data.

**Method:**

Literature searches were conducted in March 2023 using five databases to identify observational studies that used national primary care data from individuals aged 15–49 years. Preconception indicators were defined as medical, behavioural, and social factors that may impact future pregnancies; health outcomes included those that may occur during and after pregnancy.

**Results:**

From 5259 screened records, 42 articles were included. The prevalence of 37 preconception indicator measures was described for female patients, ranging from 0.01% for sickle cell disease to >20% for each of advanced maternal age, previous caesarean section (among those with a recorded pregnancy), overweight, obesity, smoking, depression, and anxiety (irrespective of pregnancy). Few studies reported indicators for male patients (*n* = 3) or associations with outcomes (*n* = 5). Most studies had a low risk of bias, but missing data may limit generalisability of the findings.

**Conclusion:**

The findings demonstrated that routinely collected UK primary care data could be used to identify patients’ preconception care needs. Linking primary care data with health outcomes collected in other datasets is underutilised, but could help to quantify how optimising preconception health and care could reduce adverse outcomes for mothers and children.

## Introduction

Preconception care is the provision of biomedical, behavioural, and social interventions to people of reproductive age (15–49 years) before conception may occur, with the aim of improving short- and longer-term parental and child health outcomes.^1^ Primary care teams have a key role in providing preconception care, as identified by patients and healthcare professionals.^2^^,^^3^ Preconception care delivered in primary care improves knowledge and preconception health behaviours in women, but there is currently less evidence about men or the impact on pregnancy and longer-term health outcomes.^4^^,^^5^ In line with the National Institute for Health and Care Excellence’s (NICE’s) Clinical Knowledge Summary on preconception advice and management, primary care teams are encouraged to consider discussions about preconception health (when appropriate) and to assess, manage, and, potentially, optimise a range of physical and mental health conditions, health behaviours, and social needs prior to potential pregnancy;^6^ however, routine provision of preconception care is not currently widespread in UK clinical practice.^7^

To build the case for implementing strategies and guidelines that optimise the population’s preconception health, the UK Preconception Partnership proposed an annual report card to describe and monitor preconception health.^8^ The authors’ scoping review^9^ to inform national surveillance identified 65 preconception indicators (that is, medical, behavioural, and social risk factors that may impact potential future pregnancies among individuals of reproductive age) that are recorded in existing UK routine health data. A first report card was produced based on 23 indicators recorded in the national Maternity Services Data Set (MSDS), demonstrating that nine in ten women in England enter pregnancy with at least one potentially modifiable risk factor for adverse pregnancy and birth outcomes.^10^^,^^11^ Similarly, an analysis of primary care data from the Royal College of General Practitioners Research and Surveillance Centre (RCGP RSC) found that 91% of women of reproductive age have a behavioural or medical risk factor for adverse pregnancy outcomes.^12^ These studies have, to date, focused on preconception health of women (not men), but have not examined trends and trajectories in medical, behavioural, and social indicators during the years leading up to pregnancy; doing so would improve the ability to identify the population’s preconception care needs throughout their reproductive years.

**Table:** How this fits in

The provision of preconception care is not currently embedded into routine clinical practice. This systematic review demonstrates that UK primary care data can provide information on the prevalence of a range of medical, behavioural, and social factors among female patients of reproductive age, while limited research has examined male preconception health. Routinely recorded data from electronic patient records can be used by primary healthcare professionals to search for preconception risk factors and support individualised preconception care; aggregate data can be used by public health agencies to promote population-level preconception health. Data-quality improvements and linkage of routine health datasets need further investment.

Routinely collected primary care data are, potentially, a unique resource to describe and monitor preconception health, and to examine the impact of (changes in) preconception indicators on improving outcomes, such as gestational diabetes and preterm birth. In order to inform future research and surveillance in the UK, and develop policy and clinical practice recommendations, the authors aimed to systematically review the literature to:

- explore how UK routine primary care data could provide information on the prevalence of preconception indicators; and
- examine associations with maternal and offspring health outcomes.

## Method

### Search strategy and selection criteria

The protocol for this review was registered with PROSPERO,^13^ and the Preferred Reporting Items for Systematic reviews and Meta-Analyses 2020 guideline^14^ was used to ensure transparent reporting. A search strategy was developed, and searches were conducted on 27 March 2023 (from inception date) using five databases: MEDLINE (Ovid), Embase (Ovid), Scopus, CINAHL, and Web of Science. The search strategies for each database are given in Supplementary Table S1. Supplementary searches using ‘preconception’ and ‘prepregnancy’ terms were conducted using UK primary care datasets and the *British Journal of General Practice* archive, as outlined in a previous article^13^ by the authors. Reference lists of included articles were screened for additional studies. Articles were selected if they:

- included findings from an observational study among individuals of reproductive age (15– 49 years);
- used national patient-level routine primary care data collected in England, Wales, Scotland, and/or Northern Ireland; and
- reported on the prevalence of at least one preconception indicator.

The population, intervention, comparator/control, outcome, study design (PICOS) framework that was used is outlined in Box 1.

**Box 1.** PICOS framework

**Population** Individuals of reproductive age who may or may not be(come) pregnant/conceive a pregnancy (any gender, aged 15–49 years).
**Intervention/exposure** Preconception indicators as identified in Schoenaker *et al*.^9^ Preconception indicators are defined as medical, behavioural, and social risk factors or exposures, as well as wider determinants of health that may impact potential future pregnancies among all individuals of reproductive age. Studies do not have to identify relevant factors or exposures as ‘preconception indicators’.
**Comparator/control** Not applicable.
**Outcome** Maternal health outcomes: any outcome that may occur during pregnancy (for example, gestational diabetes), delivery (for example, caesarean section), postpartum (for example, mortality), or beyond (no age limit) (for example, type 2 diabetes). Offspring health and developmental outcomes (including social/educational outcomes): any outcome that may occur during pregnancy (for example, stillbirth), delivery (for example, preterm birth), infancy (for example, neonatal intensive care unit admission), or beyond (no age limit) (for example, learning difficulty).
**Study design** Observational studies (including cohort, cross-sectional, and case–control studies).

*PICOS = population, intervention, comparator/control, outcome, study design.*

Included indicators were based on a list of 65 indicators (for example, weight) and 117 indicator measures (for example, underweight, overweight, and obesity) across 12 domains (for example, health behaviours and weight) identified from the authors’ previous scoping review.^9^ Articles not including new or original peer-reviewed results and conference abstracts were excluded.

### Selection process

Search results were collated in EndNote (version 20) and duplicates were removed, before being uploaded to Covidence software. Titles and abstracts, followed by full-text articles, were screened independently by two reviewers for inclusion. Disagreements or uncertainties were resolved through discussion.

### Data extraction and synthesis

A standardised data-extraction form was developed and piloted. Data were extracted by one reviewer and checked by a second reviewer; disagreements were resolved between the two. All extracted data on study characteristics (grouped by primary care database), prevalence of preconception indicators, and measures of association between preconception indicators and outcomes (grouped by preconception indicator) were presented in tables. To obtain population-level estimates of preconception indicators, prevalence data were extracted only if they had been reported (or could be calculated) for the overall study population of women or men of reproductive age (that is, not if reported only in sub-populations, such as patients with a specific condition or characteristic). Meta-analysis was not conducted because of the heterogeneity in preconception indicator definitions and the inclusion and exclusion criteria of study populations.

### Risk-of-bias assessment

Risk of bias was assessed for study findings on the prevalence of preconception indicators using the 10-item scale developed by Hoy *et al*^15^ rating internal and external validity. This Newcastle– Ottawa Scale was used to rate risk of bias of study findings on associations between preconception indicators and health outcomes based on seven items related to selection, comparability, and exposure/outcome.^16^ Risk of bias was assessed by one reviewer and checked by a second reviewer; disagreements were resolved between the two reviewers. Studies were classified as having low, moderate, or high risk of bias (findings on prevalence),^15^ and good, fair, or poor data quality (findings on associations);^16^ scoring guides are given in Supplementary Tables S2, S3a, and S3b. In addition, potential additional biases not captured through these risk-of-bias assessment tools were reported, including: the ability for indicator and outcome data to be accurately captured in primary care; high proportion of excluded or missing data (defined as >20.0%); and no, or limited, linkage with other routine health datasets (for studies reporting indicator– outcome associations).

## Results

**Figure 1.**
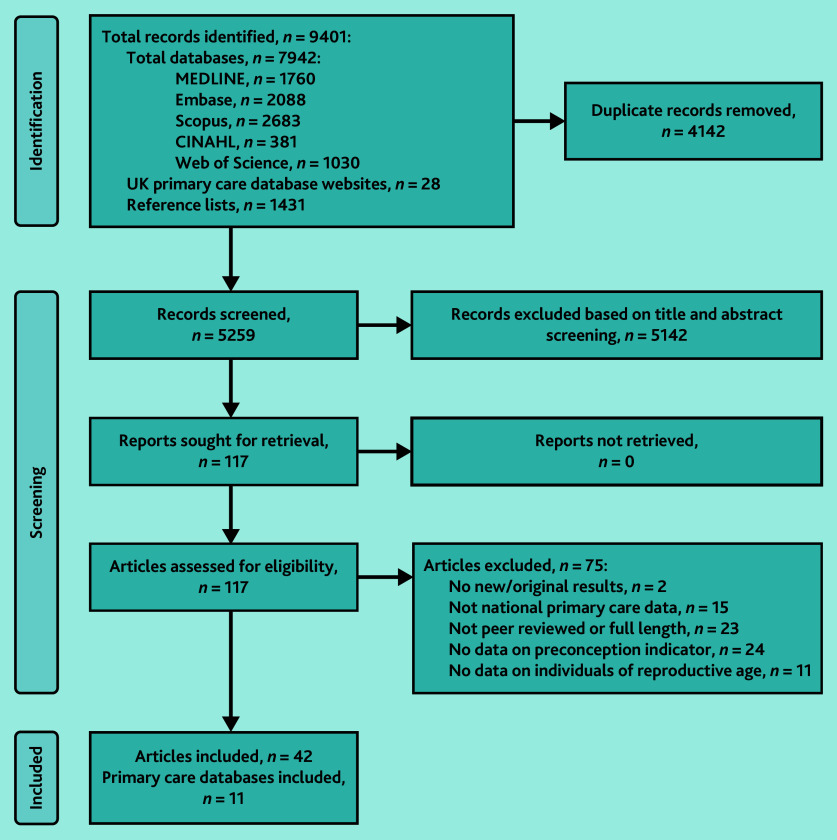
PRISMA flow diagram of the identification and selection of studies included in the review.

From 9401 identified records, 4142 duplicates were removed after title and abstract screening; of the remaining 5259 articles, 117 full-text articles were evaluated for eligibility (Figure 1). In total, 42 articles were included,^17^^–^^58^ which reported findings from 11 primary care databases, such as the Clinical Practice Research Datalink (CPRD) and The Health Improvement Network (THIN).

Articles reported findings from primary care databases that included patients from three^43^ or all four UK nations,^17^^–^^24^^,^^26^^,^^30^^–^^34^^,^^38^^–^^42^^,^^44^^–^^53^^,^^56^ or from England,^25^^,^^27^^,^^28^^,^^35^^–^^57^ Scotland,^54^^,^^55^^,^^58^ or Northern Ireland only (see Supplementary Tables S4 and S5).^29^^,^^37^ In 11 studies,^24^^,^^29^^,^^31^^,^^35^^–^^38^^,^^40^^,^^44^^,^^45^^,^^57^ a primary care dataset was linked with at least one other dataset, such as Hospital Episode Statistics (HES), the Office for National Statistics mortality register, community prescribing data, or the Avon Longitudinal Study of Parents and Children. All studies included data on women and three studies also reported preconception indicators for men.^24^^,^^28^^,^^57^

### Prevalence of preconception indicators

Articles reported findings on 25 preconception indicators (37 indicator measures) across seven domains (Table 1). Most studies included people of reproductive age, irrespective of past/future pregnancy,^19^^–^^21^^,^^25^^–^^29^^,^^32^^,^^33^^,^^37^^,^^40^^–^^46^^,^^50^^–^^56^^,^^58^ while other studies included women with a pregnancy or birth recorded during the study period,^17^^,^^18^^,^^22^^,^^23^^,^^30^^,^^31^^,^^34^^–^^36^^,^^38^^,^^39^^,^^47^^–^^49^^,^^57^ or women with a recorded pregnancy and their partners (see Supplementary Table S6).^24^

**Table 1.** Range of prevalence estimates of preconception indicators reported for people of reproductive age in UK routine primary care data

**Preconception indicator**	**Preconception indicator measure^a^**	**Studies, *n*^b^**	**Prevalence range reported across included studies, %^c^**
**Domain: wider determinants of health**			
Deprivation	Percentage of women living in the area of greatest socioeconomic deprivation (based on quintiles)	6^17^^,^^25^^,^^31^^,^^34^^,^^36^^,^^37^	13.6–22.6
Ethnicgroup	Percentage of women from a minority ethnic background	5^23^^,^^30^^,^^31^^,^^35^^,^^36^	12.8–20.0

**Domain: reproductive health and family planning**			
Maternalage	Percentage of women (with a birth recorded during the study period) aged ≤19 years at time of childbirth — teenage pregnancy	3^31^^,^^34^^,^^36^	3.1–6.7
	Percentage of women (with a birth recorded during the study period) aged ≥35 years at time of most recent pregnancy — advanced maternal age	3^31^^,^^34^^,^^36^	15.1–27.0
Obstetrichistory	Percentage of women (with a birth recorded during the study period) with a previous caesarean delivery	1^34^	23.7
Fertilityproblems	Percentage of women with a history of fertility problems	2^26^^,^^31^	1.2–3.8
Contraception	Percentage of women who use contraception (specific individual methods of contraception)	7^19^^,^^20^^,^^21^^,^^25^^,^^29^^,^^32^^,^^33^	0.1–70.1

**Domain: health behaviours and weight**			
Folic acid supplementation	Percentage of women prescribed folic acid supplements	1^25^	0.3
Weight	Percentage of women in the underweight BMI category (<18.5 kg/m^2^)	1^31^	3.9–5.9
	Percentage of women in the overweight BMI category (25.0–29.9 kg/m^2^)	1^31^	26.0–26.1
	Percentage of women in the obesity BMI category (≥30.0 kg/m^2^)	1^31^	23.2–24.6
Smoking	Percentage of women who currently smoke	3^25^^,^^31^^,^^34^	13.1–26.7

**Domain: immunisation and infections**			
Sexually transmitted disease	Percentage of women diagnosed with gonorrhoea	1^25^	0.01

**Domain: mental health conditions**			
Mental health condition	Percentage of women with any mental illness	1^30^	19.8
	Percentage of women with depression	3^17^^,^^24^^,^^31^	9.3–24.1
	Percentage of men with depression	1^24^	9.2
	Percentage of women with anxiety	2^17^^,^^31^	4.1–23.1
Serious mental illness	Percentage of women with severe mental illness	2^17^^,^^31^	0.1–2.4

**Domain: physical health conditions**			
Epilepsy	Percentage of women with epilepsy	1^31^	1.3–1.4
Diabetes mellitus	Percentage of women with type 1 diabetes	3^22^^,^^23^^,^^31^	0.2–0.6
	Percentage of women with type 2 diabetes	4^22^^,^^23^^,^^27^^,^^31^	0.2–1.1
	Percentage of women with diabetes (any type)	2^22^^,^^27^	0.6–1.4
	Percentage of women with poor diabetes control (HbA1c ≥8.5%) (among patients with type 1 diabetes)	1^27^	40.8–50.0
	Percentage of women with poor diabetes control (HbA1c ≥8.5%) (among patients with type 2 diabetes)	1^27^	24.7–33.1
Polycystic ovary syndrome	Percentage of women with polycystic ovary syndrome	3^25^^,^^31^^,^^35^	0.2–6.5
Endometriosis	Percentage of women with endometriosis	2^25^^,^^31^	0.1–1.7
Eatingdisorder	Percentage of women with an eating disorder	1^31^	1.8–1.9
Thyroiddisease	Percentage of women with thyroid disease	2^25^^,^^31^	0.1–3.3
Hypertension	Percentage of women with hypertension	1^31^	0.7–0.9
Thromboembolism	Percentage of women with thromboembolism	1^31^	0.6–0.7
Asthma	Percentage of women with asthma	1^31^	14.6–17.2
Inflammatory bowel disease	Percentage of women with inflammatory bowel disease	2^31^^,^^53^	0.5–0.6
Sickle cell disease	Percentage of women with sickle cell disease	1^31^	0.01
Cancer	Percentage of women with previous cancer diagnosis	1^31^	0.5–0.6

**Domain: medication**			
Medication not	Percentage of women prescribed valproate	1^28^	0.2–0.3
recommended when	Percentage of men prescribed valproate	1^28^	0.4
planning pregnancy	Percentage of women prescribed antidepressant medication	1^37^	16.3

a *Preconception indicator measures are based on those identified in Schoenaker* et al.^9^ *Definitions of indicator measures may differ slightly across studies (as outlined in Supplementary Table S7).*

b *Some studies include multiple primary care databases or multiple time points.*

c *Full details on the prevalence of preconception indicators reported in each study can be found in Supplementary Table S7. BMI = body mass index. HbA1c = glycated haemoglobin.*

Data on overall prevalence were available for 21 of the 42 studies,^17^^,^^19^^–^^37^^,^^53^ with the other 21 studies reporting prevalence estimates only in sub-populations. Additional preconception indicators reported in sub-populations included housing, domestic abuse, routine GP check-up in the previous year, paternal age, previous pregnancy loss, history of assisted reproduction, alcohol consumption, substance misuse, cervical screening, and cardiovascular disease (Supplementary Table S5).

The prevalence of preconception indicators reported across studies and primary care databases varied widely, possibly due to differences in preconception indicator definitions, year of data collection (Supplementary Table S7), and study populations (Supplementary Table S6). The prevalence of preconception indicators defined in line with the authors’ scoping review (that is, excluding individual methods of contraception and prescribed folic acid supplements)^9^ ranged from 0.01% for sickle cell disease^31^ and gonorrhoea^25^ to >20% in the majority of studies for each of advanced maternal age,^31^^,^^34^^,^^36^ previous caesarean section (among those with a recorded pregnancy),^34^ poor diabetes control,^27^ overweight,^31^ obesity,^31^ smoking,^25^^,^^31^ and diagnosis of depression and anxiety among women (irrespective of pregnancy).^24^^,^^31^ Only three studies reported preconception indicators for men;^24^^,^^28^^,^^57^ they showed, for example, that the prevalence of depression among fathers (9.2%) was lower than that for with mothers (22.2%),^24^ and that the proportion of patients prescribed valproate was comparable among women (0.31%) and men (0.37%) in 2004, but much lower among women (0.16%) than men (0.36%) in 2018.^28^

### Associations of preconception indicators with maternal and offspring outcomes

Five studies reported associations of preconception indicators (contraception prescription,^39^ sexually transmitted disease,^25^ and polycystic ovary syndrome^35^^,^^38^^,^^40^) with pregnancy and birth outcomes (Supplementary Table S8). Outcome data were obtained from primary care data and/or linked HES data. Where two studies reported on comparable indicators and outcomes, consistent findings were shown for associations of polycystic ovary syndrome with preterm delivery (<37 weeks’ gestational age) (positive association), high birthweight (>4 kg) (no association), and low birthweight (<2.5 kg) (inconclusive findings).^35^^,^^40^

### Risk of bias and data quality

Risk of bias for findings relating to the prevalence of preconception indicators was generally low (*n* = 17/21 studies);^17^^,^^19^^–^^23^^,^^25^^–^^30^^,^^32^^–^^34^^,^^37^ however, none of the studies received a minimal score (no bias) (Supplementary Table S2). Potential biases were introduced based on representativeness and sampling frame (for example, excluding women with no pregnancy reported or no linked data available), and indicator definition and measurement (for example, reporting individual methods of contraception rather than population-prescribed contraception, or reliance on medication prescription rather than dispensing data).

Studies included in this review documented substantial missing data, ranging from approximately 20%–60% for ethnicity and body mass index category, likely varying across sub-populations. Moreover, details of non-response (for example, impact of missing data) were not reported in approximately half the studies,^17^^,^^19^^,^^20^^,^^24^^,^^29^^,^^33^^,^^53^ and data on some indicators are not accurately captured in primary care data (for example, use of over-the-counter 400 mcg folic acid supplements).

Data quality was rated ‘good’ for four of the five studies that examined associations of preconception indicators with health outcomes (Supplementary Tables 3a and 3b).^35^^,^^38^^–^^40^

## Discussion

### Summary

This systematic review found that UK routine primary care data can provide valuable information on patients’ medical, behavioural, and social risk factors before (a potential) pregnancy. Based on 42 included studies among people of reproductive age or women with a pregnancy recorded during the study period, the prevalence of 37 preconception indicator measures was reported. Findings showed that >20% of women of reproductive age could potentially benefit from support regarding smoking cessation, weight management, and management of depression and anxiety; this could not only optimise their own health, but could also improve their chance of a successful pregnancy and healthy baby if desired.

Limited research has used primary care data to examine preconception indicators among men, or associations of preconception indicators with pregnancy outcomes and longer-term maternal and offspring health outcomes.

### Strengths and limitations

To the authors’ knowledge, this is the first systematic review to demonstrate how national routine primary care databases can be used to describe the population’s preconception health, and to inform clinical practice and future research directions. Comprehensive, prospectively registered review methods were used. The authors’ search was limited to national primary care data from the UK, as routinely collected electronic patient record (EPR) data and their availability and use for research purposes may differ across countries; the findings would, therefore, not have identified preconception indicators reported in any specific local datasets and may not be generalisable to other countries.

Preconception indicators were selected based on the authors’ previous scoping review;^9^ as a result, potentially relevant indicators not included in that review, not reported in the included studies, or not used and published for research purposes would have been missed. Data completeness in terms of preconception indicators that are (and are not) reported within each primary care dataset were not reported on, as this largely relies on the types of research that have been conducted and published using individual databases, which does not necessarily reflect the availability of data. Moreover, some preconception indicators (such as dietary intake and physical activity) are not routinely recorded in general practice.

### Comparison with existing literature

Findings from this review complement the authors’ previous preconception report card^10^ based on the MSDS, showing that national routine health data are a valuable resource to describe and monitor women’s preconception health. Half of the preconception indicators identified in this review were also reported in the MSDS, with comparable prevalence estimates for most indicators (for example, teenage pregnancy, previous caesarean delivery, overweight, and obesity); other indicators may be underreported in primary care (for example, over-the-counter folic acid supplementation) or in the MSDS (for example, mental health conditions).^10^ Published primary care data reported an additional 15 indicators that were not included in the MSDS (for example, fertility problems, contraception, relevant medical conditions, and teratogenic medication use).^19^^–^^21^^,^^25^^,^^26^^,^^28^^,^^29^^,^^31^^–^^33^^,^^35^^,^^37^ Based on linkage of primary care and HES datasets, findings from the review presented here (regarding two studies^35^^,^^40^) confirm the previously reported association of polycystic ovary syndrome with increased risk of preterm delivery.^59^

Findings from this review are also in line with previous research^60^^–^^62^ that has reported issues with the quality of data in primary care. Coding quality is related to financial incentives, such as the Quality and Outcomes Framework; this may improve the accurate recording of selected indicators, but may also distort prevalence estimates over time.^60^ The prevalence of some preconception indicators may be underestimated, as not all conditions are solely diagnosed and coded in general practice (for example, sexually transmitted disease),^25^ or medications and supplements prescribed (for example, contraception and 400 mcg folic acid supplements).^63^ Another commonly reported limitation is the representation of selected general practices in research databases, often limited to practices that use one of four main software platforms to manage EPRs and further determined by voluntary ‘opt ins’.^60^^,^^61^ As a result, primary care databases may underrepresent specific regions and bias national prevalence estimates of preconception indicators and associations with health outcomes.

### Implications for research and practice

Collectively, findings from the studies included in this review demonstrate that routinely collected primary care data in the UK can provide valuable information on patients’ medical, behavioural, and social risk factors before (a potential) pregnancy. These data can guide the provision of individualised preconception care and be used as a valuable resource for research and surveillance.

The findings presented here demonstrate that many preconception indicators are routinely recorded in EPRs, allowing primary healthcare professionals to search for risk factors and provide individualised preconception care. A digital risk screening template has already been developed, based on the NICE Clinical Knowledge Summary^6^ and using software from Ardens (https://ardens.org.uk), which is a clinical decision support system and UK-wide provider of digital templates and resources for >3300 GP practices. This template can be used as a tool to bring together and analyse EPR data to help primary healthcare professionals identify and address individual patient preconception risk factors, make informed decisions, and, thereby, improve personalised patient care. The template may also improve the coding and recording of indicators. Further work is required to co-develop practical guidance and resources to support the integration of preconception care and use of the digital risk screening template into everyday clinical practice.

This review’s findings identify the need to use standardised definitions when reporting preconception indicators. Because of heterogeneity in definitions, the prevalence of preconception indicators across UK nations and changes over time could not be directly compared across studies; however, Lee *et al*^31^ applied standardised definitions to CPRD (UK) and Secure Anonymised Information Linkage (SAIL) data (Wales), showing comparable prevalence estimates for some indicators (for example, obesity and depression), but higher (for example, smoking, underweight, anxiety, and asthma) or lower (for example, advanced maternal age) prevalence for other indicators, when comparing pregnant women in Wales with those in the UK overall. Moreover, standardised reporting within the same database showed, for example, increases over time in the prevalence of type 2 diabetes (1995–2012) in the THIN database^23^ alongside decreases in poor diabetes control (2004–2017) in the RCGP RSC database.^27^

The limited reporting of preconception indicators in men, and associations of preconception health with pregnancy along with maternal and offspring health outcomes, calls for further research. Many of the preconception indicators reported for women are also relevant to men (for example, smoking and obesity); increasing evidence suggests that better paternal preconception health is associated with reduced risks of infertility, as well as adverse pregnancy and offspring health and developmental outcomes.^64^^–^^66^ To enable further research, improvements are needed in the way that families (that is, biological parents and their children) can be identified and data are linked, as highlighted by Davé *et al*^24^ and Lut *et al.*^67^

Primary care data also provide a unique opportunity to examine trajectories of preconception health during reproductive years, irrespective of pregnancy, and to quantify the extent to which these reduce adverse pregnancy and offspring health outcomes. Future research would be enhanced by linking primary care and other routine health datasets beyond the identified existing linkages (for example, MSDS and Community Services Data Set) to determine the short- and longer-term benefits of preconception care.

Recommendations on how the use of UK routine primary care data could be improved for clinical practice, research, and surveillance of preconception health and care are summarised in Box 2.

**Box 2.** Recommendations to improve the use of UK routine primary care data for clinical practice, research, and surveillance of preconception health and care

Develop coding practice standards with appropriate incentives to improve data quality. Standardise the reporting of preconception indicators, as well as pregnancy and offspring health outcomes — for example, through the development of core outcome sets. Improve the coding and identification of family and household members to enable linkage of data from biological parents and their children. Ensure nationwide linkage of general practice systems and linkage of primary care datasets with other routine health datasets (such as Hospital Episode Statistics, Maternity Services Data Set, and Community Services Data Set).
